# Migration of Silicone Particles Into the Liver Following Rupture of Silicone Breast Implant: A Case Report and Literature Review

**DOI:** 10.7759/cureus.101747

**Published:** 2026-01-17

**Authors:** Myat Win, May Su Hlaing, Win Htet, Nay Phone Hlyan, Mahmoud Mostafa, Shabbir Poonawala

**Affiliations:** 1 General Surgery, Mersey and West Lancashire Teaching Hospitals NHS Trust, Prescot, GBR; 2 General Internal Medicine, United Lincolnshire Teaching Hospitals NHS Trust, Boston, GBR; 3 General Surgery, United Lincolnshire Hospitals NHS Trust, Boston, GBR; 4 Breast Surgery, Wirral University Teaching Hospital NHS Foundation Trust, Wirral, GBR

**Keywords:** breast implant, hepatic granuloma, silicone implant rupture, silicone oil migration, siliconoma

## Abstract

Breast augmentation using a silicone implant is a popular procedure. However, there are rare complications being reported in addition to common risks following breast implant insertion. Our aim is to report one of the rare complications, migration of silicone particles into the liver, following rupture of the silicone breast implant. The patient previously had insertion of bilateral silicone breast implants. She was presented with pain and swelling of the left breast for which she had mammograms, ultrasound scans of the breasts, and magnetic resonance imaging (MRI) of the breasts. They identified rupture of the silicone implant on the left side and incidentally detected silicone granulomas in the liver on an MRI scan of the breasts. These hepatic lesions were then confirmed by a CT scan of the liver. She underwent the removal of bilateral breast implants. However, she was asymptomatic of hepatic silicone granulomas, and no further treatment was required. Since it is an unusual complication, we conducted a review of the literature describing the mechanisms of silicone migration, diagnosis, and management of this silicone granuloma. Our report is the first report from the UK to our knowledge. We identified four more cases reported from the USA, Colombia, and Italy. In conclusion, the diagnosis of silicone granuloma in the liver is challenging since clinical features are occult, and they mimic metastatic deposits on imaging. Its diagnosis and management necessitate input from a multidisciplinary team. Clinicians should maintain a high index of suspicion of silicone migration in patients who present with unexplained hepatic lesions and have had a breast implant inserted in the past.

## Introduction

Breast augmentation with implants has been performed since 1962. It remains the most common cosmetic surgery in the UK, with about 6,700 procedures in 2022 according to the British Association of Aesthetic Plastic Surgeons [1]. However, the procedure carries its own risks. According to the multi-centred cohort study, which reported complications of breast implants in 99,993 patients, 56% of implants were made with silicone. It was reported that saline implants ruptured more than silicone implants (2.5% vs. 0.5%), whereas silicone implants were found to have more significant capsular contracture (5.0% vs. 2.8%) [2]. When saline implants rupture, breasts may flatten suddenly. However, the cohesive property of silicone gel maintains breast shape, and it can be unnoticeable on physical examination when the silicone implant ruptures or leaks. Therefore, the FDA has recommended periodic imaging to detect silent rupture of silicone breast implants [3]. 

Silicone has been widely utilized in medical devices, including intraocular lenses, artificial heart valves, testicular prostheses, joint replacements, and, most notably, breast implants [4]. Although historically regarded as biologically inert, growing evidence highlights silicone’s potential to elicit both local and systemic biological responses [5,6]. Silicone from ruptured implants or gel bleed can migrate through both lymphatic and blood pathways, depositing in multiple organs, including lymph nodes, lungs, spleen, liver, and distant subcutaneous sites [7]. While subcutaneous siliconomas are common, silicone involvement of the liver is rare but clinically important [8].

In this paper, we present another rare case of silicone granulomas in the liver, also known as hepatic siliconomas, following a rupture of the breast implant. Our case is the first one reported from the United Kingdom, to our knowledge. Only four cases from Colombia, the USA, and Italy have been described in the published literature, highlighting the rarity of this condition [8-10]. We carried out a narrative review of studies describing silicone migration mechanisms, hepatic histopathology, and diagnostic approaches, aiming to raise awareness of this under-recognized complication.

## Case presentation

A 53-year-old lady was referred to our breast surgical clinic in March 2024 due to symptoms such as swelling and pain in the left breast for four weeks. She denied any nipple discharge or injury to the left breast. She had bilateral breast augmentation done using implants and mastopexy 12 years ago at the age of 40. She did not have any immediate post-operative complications. She was fit and well without having any other medical problems. She has been post-menopausal since the age of 50 and has hada Mirena coil in situ since 2019. She has been using Estradiol HRT gel. She neither drinks nor smokes.  

The examination revealed that the left breast looked bigger than the right one. However, there was no sign of inflammation, and no discrete mass was palpable in the left breast. The right breast implant felt smooth, and no discrete mass was palpable in the right breast. Both breasts revealed well-healed scars around the areola. Both axillary and supra-clavicular areas felt fine.

**Figure 1:**
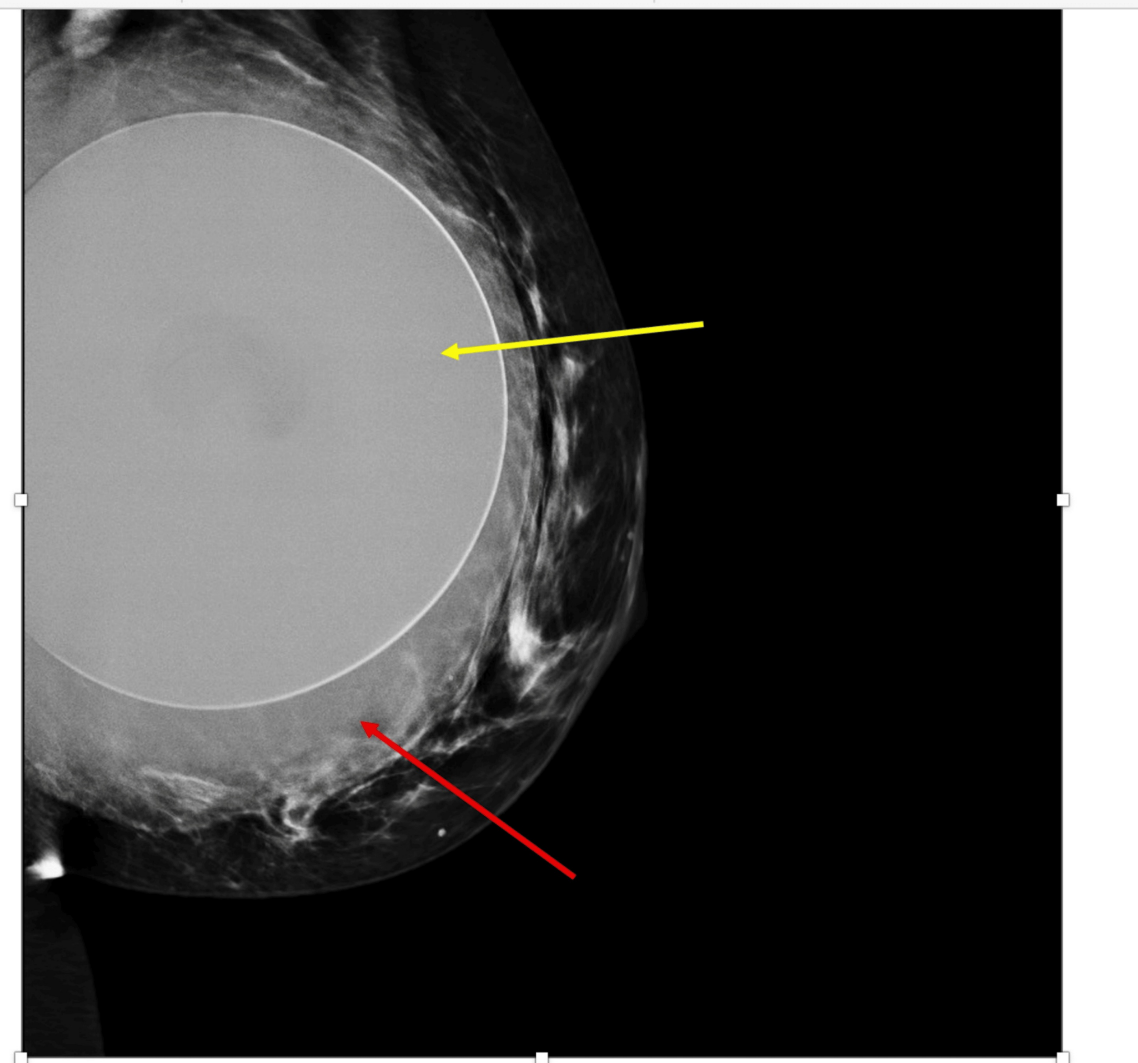
Left mammogram revealed a sub-glandular implant with a smooth outline (yellow arrow) and a further low-density collection around the left implant (red arrow).

She initially had bilateral mammograms, ultrasound scans of the left breast and axilla in March 2024. The left mammogram revealed a sub-glandular implant with a collection around the implant (Figure 1). There were no suspicious findings seen in either breast. 

**Figure 2:**
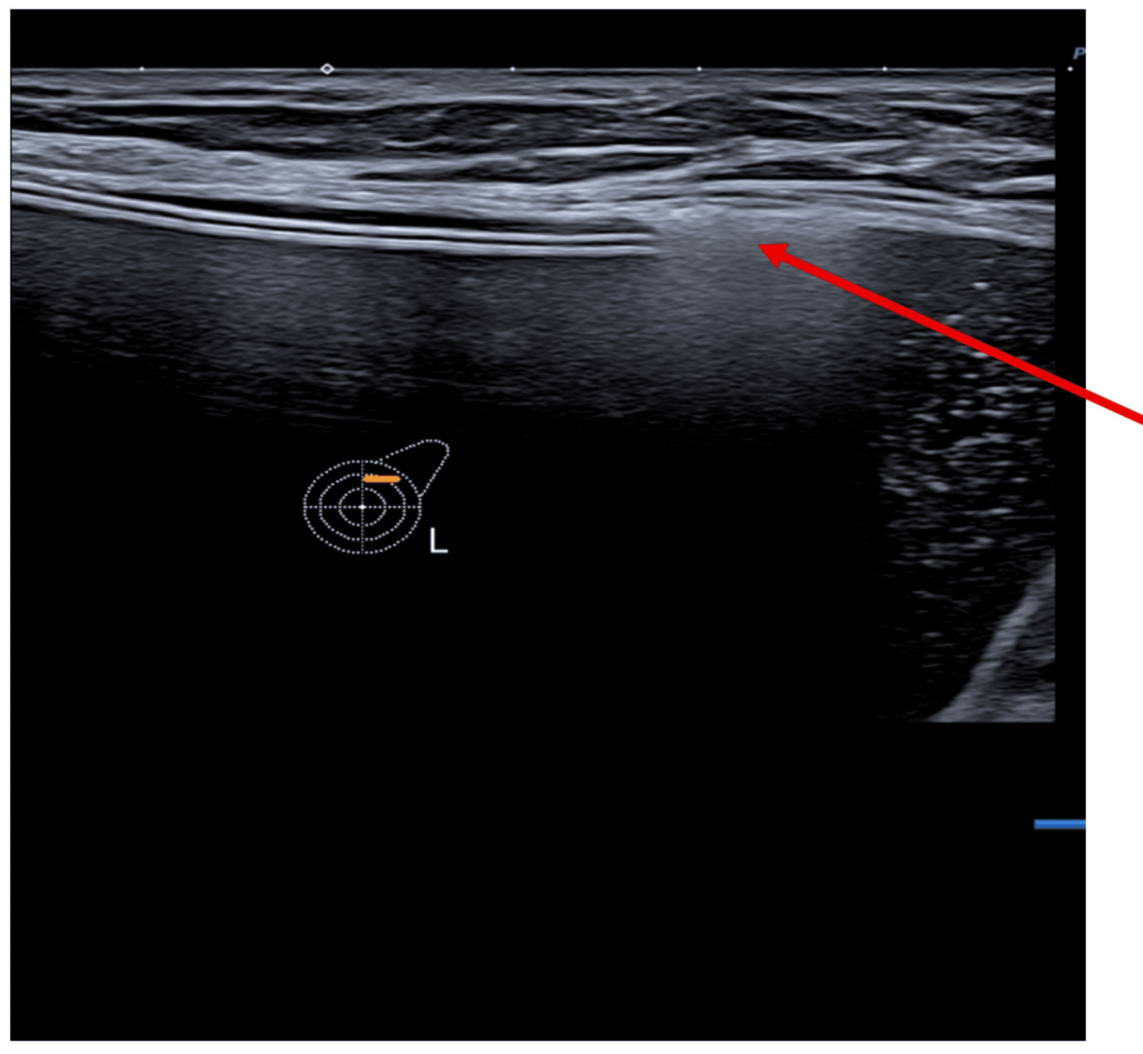
Ultrasound scan of the left breast revealed a single lumen implant with peri-implant echogenicity representing the intracapsular rupture (arrow).

**Figure 3:**
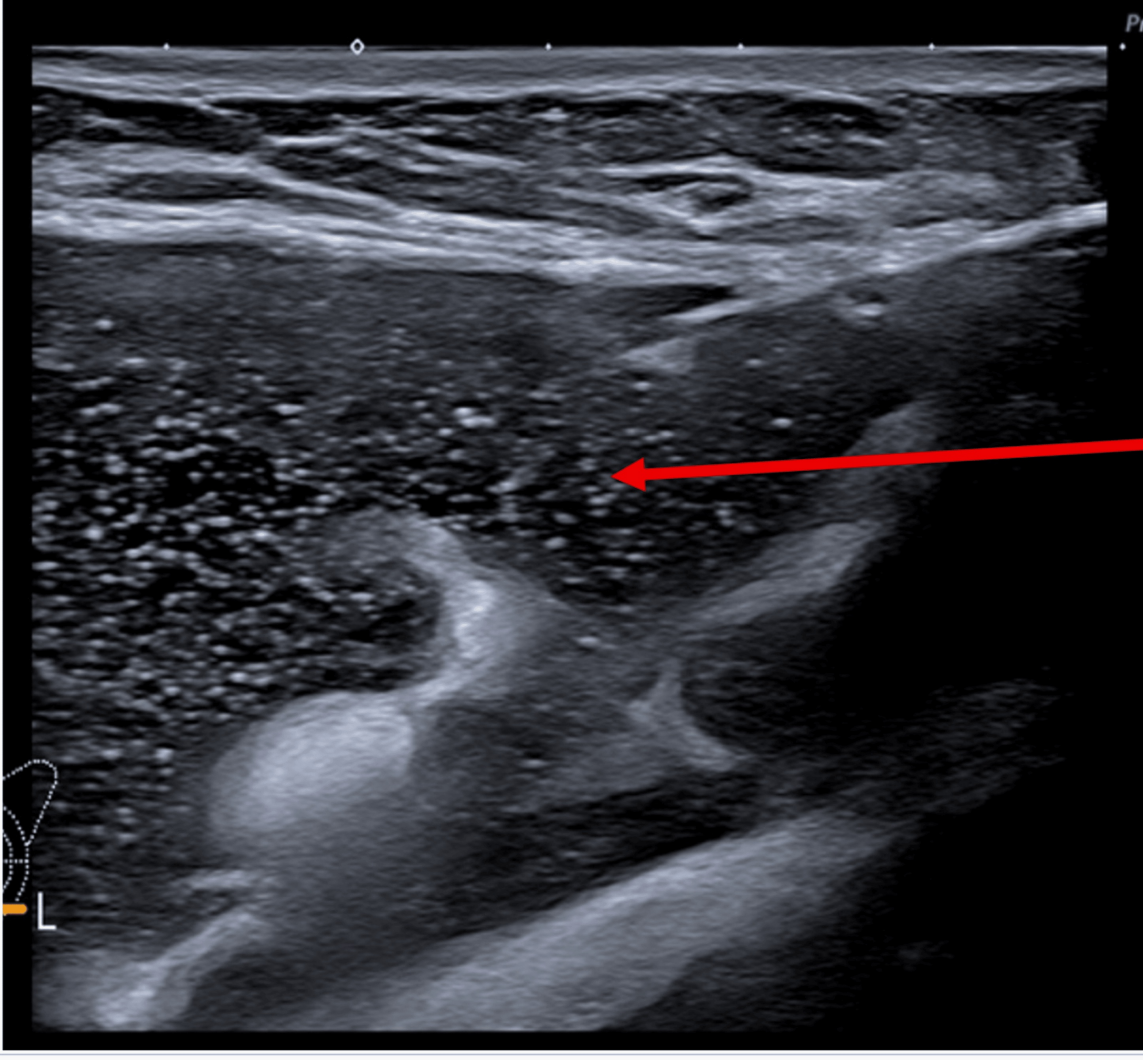
Ultrasound scan of the left breast revealing a large peri-implant collection with echogenic particles within the collection (arrow).

**Figure 4:**
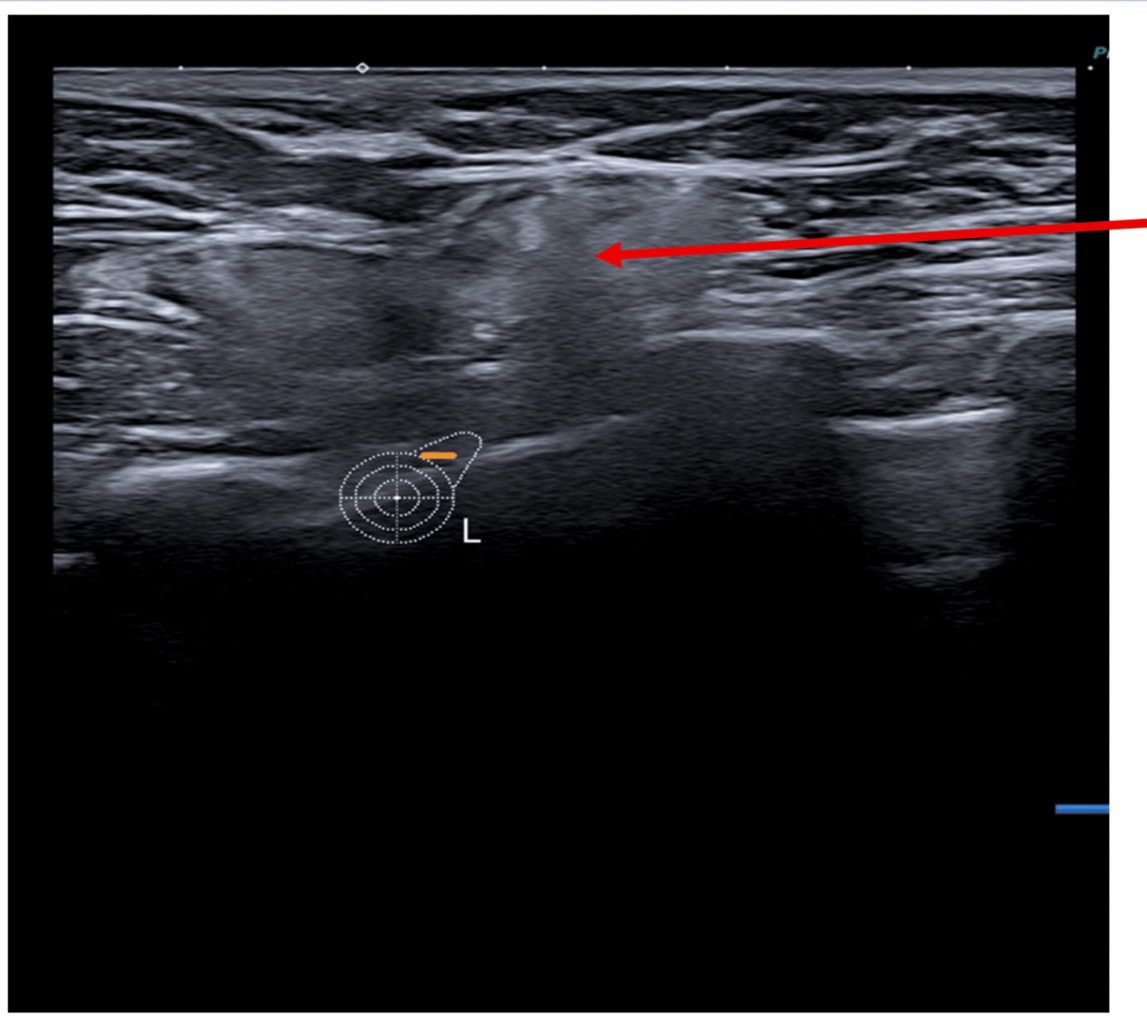
The ultrasound scan of the left axilla revealed a snowstorm appearance (arrow) since lymph nodes were loaded with silicone.

The ultrasound scan (USS) of the left breast reported intracapsular rupture (Figure 2). It also showed a large peri-implant collection (Figure 3). The USS of the left axilla revealed lymph nodes loaded with silicone (Figure 4). 

**Figure 5:**
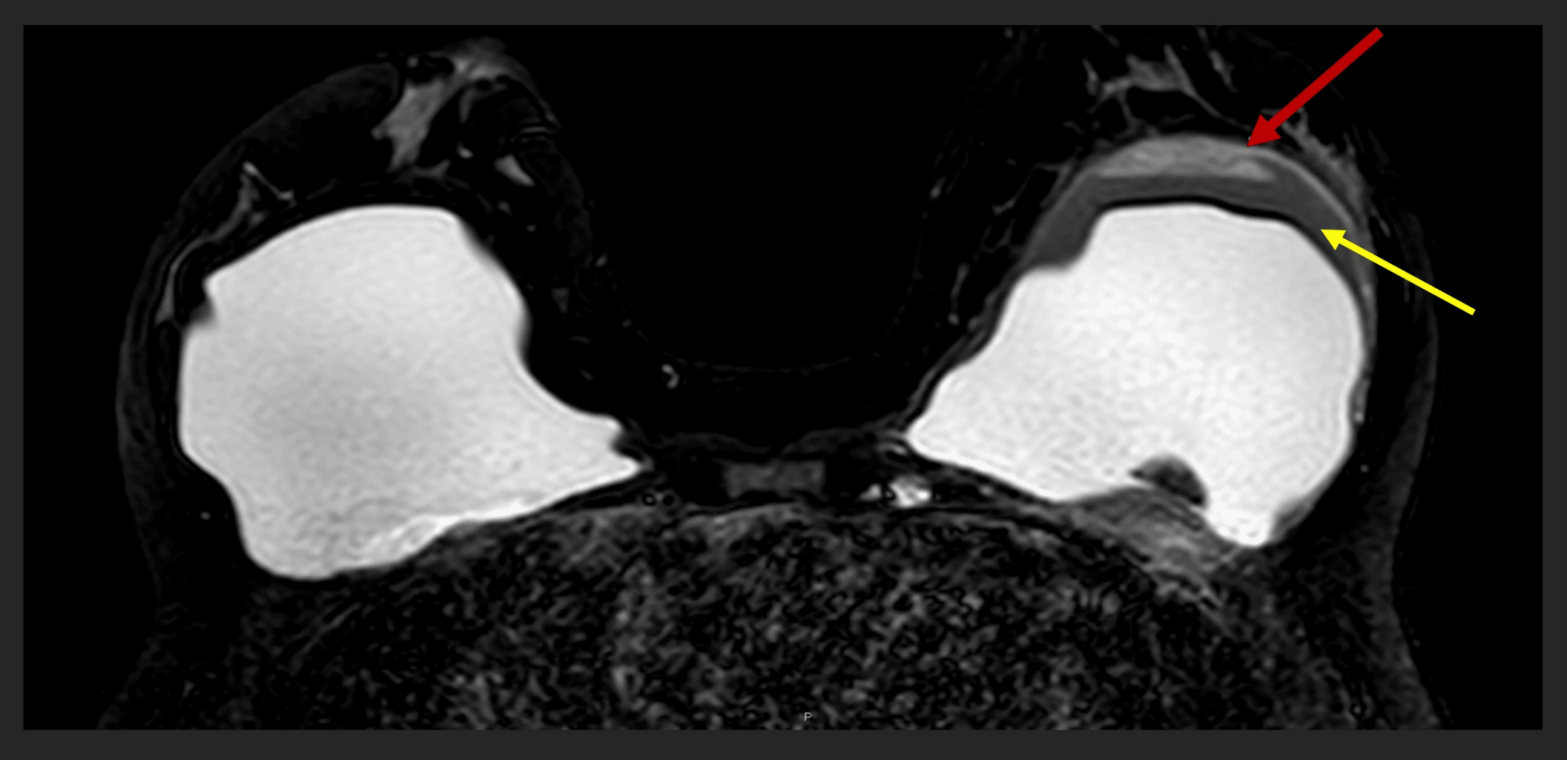
MRI breasts revealed intra-capsular rupture with silicone (red arrow) floating in the peri-implant effusion (yellow arrow) on the left breast. MRI: magnetic resonance imaging

**Figure 6:**
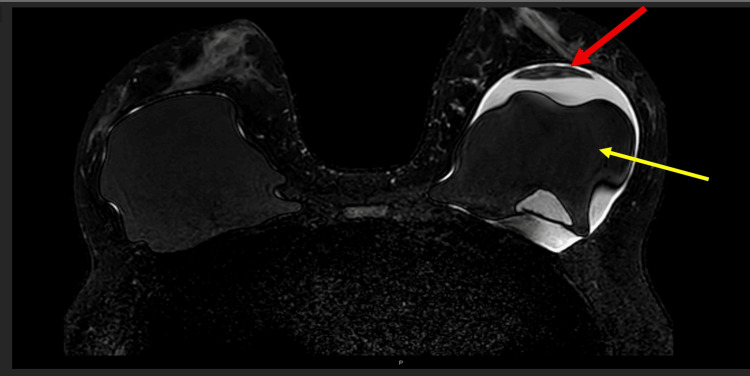
The left silicone implant (yellow arrow) and floating piece of silicone (red arrow) appeared dark in the silicone-suppressed MRI scan of the breasts. MRI: magnetic resonance imaging

She underwent magnetic resonance imaging (MRI) of both breasts with contrast to rule out implant-related large cell anaplastic lymphoma and to assess the integrity of the implant as per the implant protocol in June 2024. There was no evidence of malignant enhancement identified on imaging. However, it confirmed intra-capsular rupture with silicone floating in the peri-implant effusion (Figure 5). It could also be clearly seen on the silicone-suppressed MRI scan, where the silicone implant and the floating piece of silicone appeared dark (Figure 6). There was no definite extra-capsular rupture.

**Figure 7:**
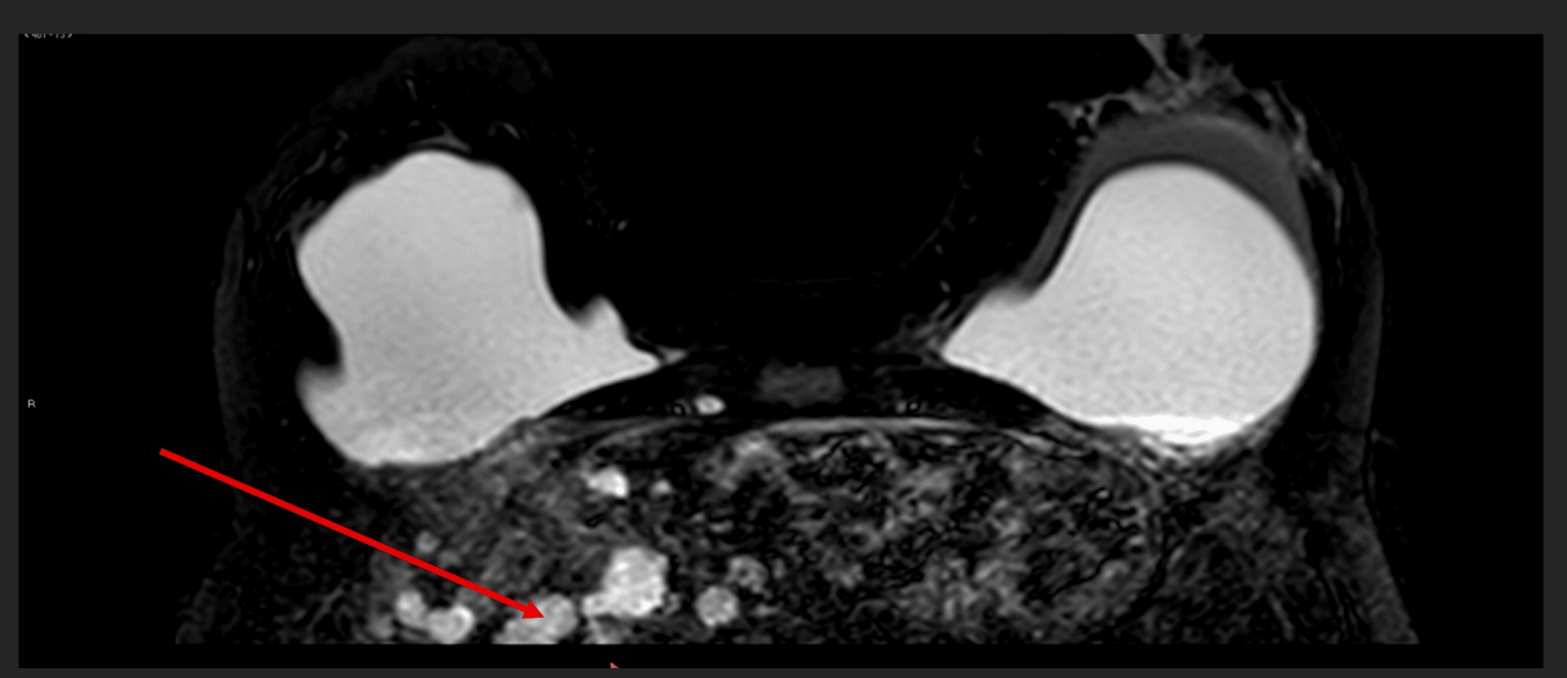
MRI scan of breasts detected silicone containing granulomata (arrow) within the liver incidentally. MRI: magnetic resonance imaging

**Figure 8:**
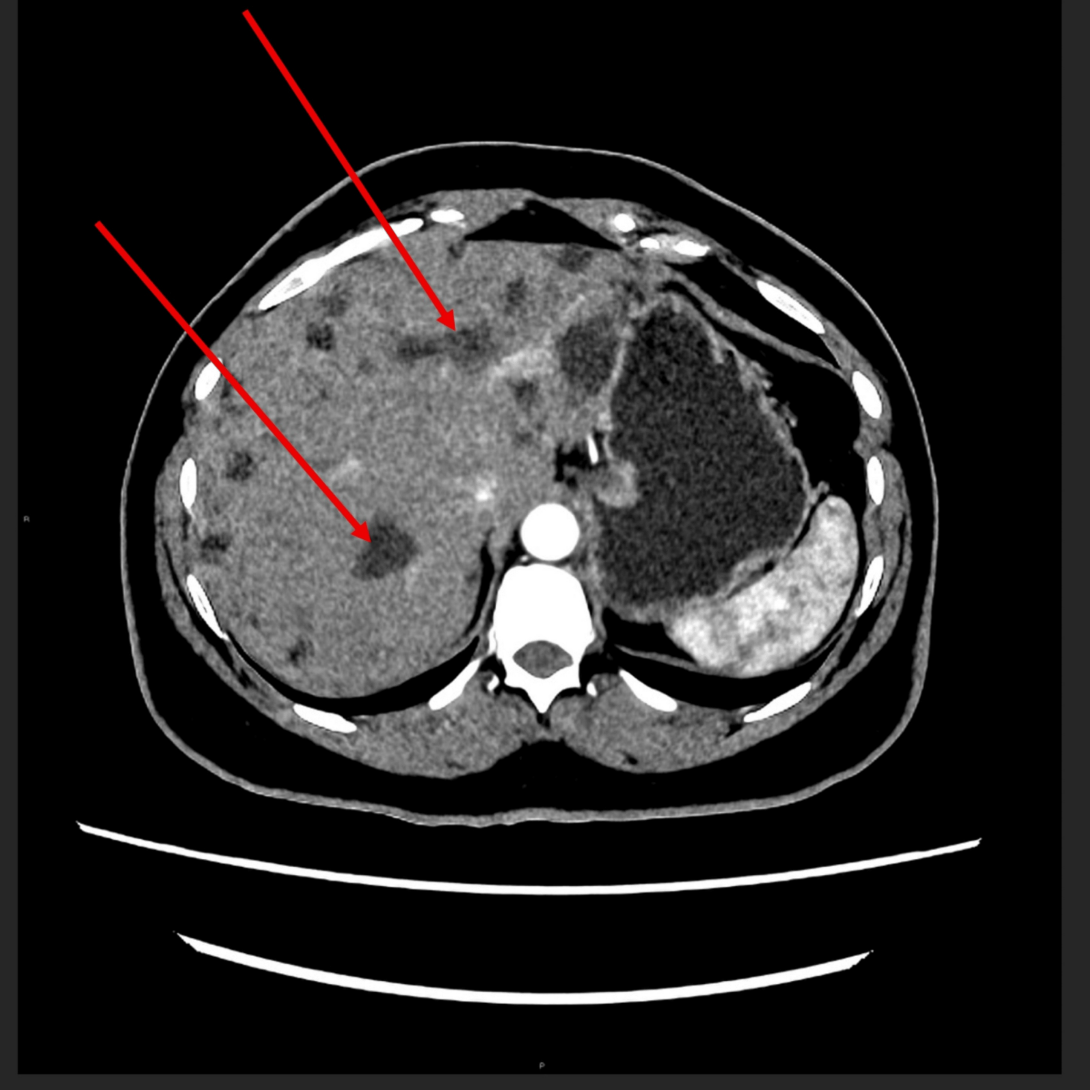
Multiple hypodense hepatic lesions (arrows) were revealed on the CT scan of the liver (arterial phase) CT: computerized tomography

MRI scan also incidentally picked up multiple silicone-containing granulomata within the liver (Figure 7). She then had a computerized tomography (CT) scan of the neck, thorax, abdomen, and pelvis to rule out anything sinister. CT scan of the liver (arterial phase) revealed multiple hypodense hepatic lesions without any enhancement (Figure 8). It is interesting to see how further investigation into the patient's breast pain and peri-implant fluid has led to the detection of hepatic involvement, which has been silent. 

The patient initially underwent ultrasound-guided drainage of fluid collection around the left implant to control her symptoms while waiting for the results of MRI and CT scans. Three hundred and fifty milliliters (350ml) of fluid was drained, and it was cloudy green. Twenty-five milliliters (25 ml) of them were sent for cytology. Cytology did not show any high-grade lymphoma or carcinoma cells, and immunostaining for CD30 revealed only a small amount of possible activated B cells. This fluid sample was sent to the regional haemato-oncology diagnostic service (HODS) as well to rule out breast implant-associated anaplastic large cell lymphoma (BI-ALCL). Sections of the cell block revealed a fibrinous material incorporating scattered small lymphoid cells, numerous histiocytes, few neutrophils, and occasional eosinophils. The large population of histiocytes was highlighted by the CD68 stain. Morphologically, there appeared to be no large anaplastic cells in the sections examined, and there appeared to be no evidence of this on the CD30 stain. A CD30 stain showed very few activated blasts. CD20 and CD3 stains showed scattered B and T lymphoid cells, respectively. In summary, there was no overt evidence of involvement by BI-ALCL, and overall features were most likely reactive changes. Full blood count, urea, electrolytes, and liver function tests were within the normal range. 

Eventually, due to symptoms such as pain and recurrent fluid collection around the implant, the patient underwent bilateral removal of implants and partial capsulectomy in mid-July 2024. Since we did not want to compromise the blood supply to the nipples, given the previous mastopexy operation, the procedure was approached via the incisions above the infra-mammary fold on both sides. Both implants were identified in the pre-pectoral planes. The right implant was found to be intact, whereas the left one was ruptured with surrounding yellowish fluid as well as whitish deposits. The capsule of the left implant was thickened. Post-operatively, the left breast implant with its capsule was extensively analysed by immunohistochemistry. The result confirmed fibrosis, degeneration, and reactive lymphoid hyperplasia without any evidence of lymphoid or epithelial neoplasia.

She was followed up at three weeks and nine months after the surgery. Both scars healed well, and there was no post-operative complication. Patient felt content with her breasts’ appearance following the removal of both implants. There was no further fluid collection in the breast following this surgery. Nipple sensation on both sides had been retained. She remained asymptomatic with hepatic granuloma clinically and biochemically. Therefore, we did not arrange a biopsy of hepatic granuloma, further blood tests, and imaging for the liver lesion after the multidisciplinary discussion. She did not require any further treatment for silicone particles in the axilla, either. She was then discharged from our care. 

## Discussion

Silicone breast implants and complications 

Silicone breast implants, first introduced in the 1960s, consist of polymers composed of repeating silicon-oxygen backbones. Depending on their physical characteristics, these materials can be manufactured into oils, gels, or elastomers. Despite their widespread use, silicones are not entirely biologically inert and have been associated with a spectrum of host responses [5,6]. Complications of breast augmentation with implants include seroma, haematoma, capsular contracture, infection, asymmetry, displacement, and rupture of the implant [2,11]. 

Mechanisms of silicone migration 

Silicone migration primarily occurs due to implant rupture, which may be intracapsular or extracapsular. In intracapsular rupture, the leaked material remains within the fibrous capsule, whereas extracapsular rupture permits spread into adjacent tissues. Migration can follow several routes, such as gravity-assisted migration along subcutaneous planes to the abdominal wall or vulva; lymphatic and hematogenous spread to organs; and microscopic leakage even in intact implants due to weakness in the outer shell, leading to silicone deposition and systemic spread [12,13]. 

Factors contributing to implant rupture include trauma, aging implants, closed capsulotomy, and surgical instrument damage [14,15]. The cohesiveness of the silicone gel is a significant determinant of its migration potential. The lower-cohesive gels leak more readily than highly cohesive formulations designed to resist dispersal [16]. 

Systemic and histological effects of silicone 

Once implanted, silicone is typically encapsulated by a fibrous membrane consisting of collagen. Leakage, either through microbleed or overt rupture, can result in migration of silicone to distant tissues. Histologically, leaked silicone appears as translucent vacuoles within fibrous tissue. In cases of extracapsular dissemination, the immune system mounts a granulomatous response, forming siliconomas that may clinically mimic neoplastic lesions. Rarely, silicone migration may mimic or even trigger conditions like Kikuchi disease [17]. Silicone can migrate to diverse tissues, including the lymph nodes, lungs, spleen, liver, and skin [7]. Systemic dissemination can also lead to regional lymphadenopathy, particularly in the ipsilateral axilla, where silicone-laden macrophages and foreign body giant cells may be identified [18]. 

Diagnosis of hepatic siliconoma 

The diagnosis of hepatic siliconoma following breast implant rupture is challenging due to its rare condition. Even identifying the implant rupture itself is not straightforward due to the high incidence of “silent” ruptures, often lacking overt clinical symptoms. MRI remains the most sensitive modality for evaluating the implant integrity, with signs such as the linguine sign indicating a collapsed implant shell and the inverted-loop sign suggesting the intracapsular rupture. For detecting silicone migration, MRI offers superior tissue contrast and multiplanar imaging capabilities. MRI can also track silicone migration from the breast to the abdominal cavity [19]. 

We reviewed previously published case reports and summarized them to help us understand their presentation, diagnosis, and management (Tables 1, 2). 

**Table 1:** Characteristics of previously reported studies.

No	Author	Year	Country	Age (years)	Source of silicone in the liver	Management of liver granuloma
1	Colapietro et al. [8]	2024	Italy	69	Ruptured breast implant	Analgesia
2	Hudacko et al. [10]	2019	USA	48	Ruptured breast implant	Lost to follow up
3	Hudacko et al. [10]	2019	USA	58	Ruptured breast implant	No treatment required
4	Posso-Osorio et al. [9]	2018	Colombia	55	Ruptured breast implant	No treatment required

**Table 2:** Clinical presentation and diagnosis of previously reported studies. CRP: C-reactive protein, ESR: erythrocyte sedimentation rate, ALP: alkaline phosphatase, GGT: gamma-glutamyl transferase, ALT: alanine aminotransferase, AST: aspartate aminotransferase, CT: computed tomography, MRI: magnetic resonance imaging

No	Author	Symptoms	Laboratory	Imaging	Histology
1	Colapietro et al. [8]	Pain in the right hypochondrium and fever	Thrombocytopenia, normal liver function test	MRI: enlarged liver volume, thickening of periportal spaces and splenomegaly	Hepatic silicone granulomas
2	Hudacko et al. [10]	Malaise and dyspnoea on exertion	Raised CRP, ESR, ALP, GGT	CT: attenuation of the liver with innumerable small, round, low-density lesions ranging from 2 mm to 2 cm. MRI: rupture of the left breast implant	Poorly formed non-necrotic granuloma. Trichrome stain - mild periportal fibrosis. Reticulin stain - nodular degenerative hyperplasia. Energy dispersive spectroscopy - presence of a small amount of silica and aluminium.
3	Hudacko et al. [10]	Nausea, chronic constipation, and weight loss	Raised ALP, ALT, GGT	CT: a small hepatic cyst	Non-necrotic foreign body giant cell type granulomas. Trichrome stain - periportal fibrosis with few fibrous septa and fibrosis around lobular granulomas.
4	Posso-Osorio et al. [9]	Pain in the right hypochondrium, hyperthermia, pruritus, chronic fatigue, and myalgia	Raised ALT, AST, and positive antimitochondrial antibodies	MRI: cholecystitis and abnormal hepatic enhancement without focal lesions	Hepatic infiltration by silicone

Clinical features 

Patients with hepatic silicone granulomas commonly present with nonspecific symptoms, making clinical suspicion difficult. Reported symptoms include fatigue, discomfort in the right upper quadrant of the abdomen, and low-grade fever. These vague presentations may delay diagnosis and are often misattributed to more prevalent hepatic conditions [8,9,10]. 

Laboratory tests

Key biochemical findings are raised alkaline phosphatase (ALP) and gamma-glutamyl transferase (GGT), indicating biliary or portal tract involvement. Transaminases (ALT and AST) remain normal or slightly elevated, pointing to a cholestatic or infiltrative pattern rather than direct hepatocellular injury [8,9,10]. 

Imaging**

Radiologically, silicone-related hepatic granulomas may mimic metastatic lesions or primary hepatic malignancy. Imaging often shows nodular lesions, hypoechoic or hyperechoic foci, or altered liver texture on ultrasound, CT, and MRI scans. These findings are nonspecific, and in many cases, definitive diagnosis requires histological confirmation via liver biopsy [8,9,10]. 

Histopathology**

Biopsy of the lesion in the liver remains the gold standard for definitive diagnosis, particularly when hepatic or deep tissue involvement is suspected. Histologically, silicone-associated hepatic injury is characterized by foreign body granulomas, often composed of multinucleated giant cells and macrophages containing variably sized vacuoles. These vacuoles represent dissolved silicone droplets, resulting in the classic "Swiss cheese-like" appearance under light microscopy [8, 10]. 

In some cases, the silicone is not apparent on routine staining, requiring electron microscopy and energy-dispersive X-ray spectroscopy (EDS) for identification. The histopathological spectrum can vary significantly. Some cases show chronic active hepatitis with portal and lobular inflammation, nodular fibrosis, and bile duct injury. Others demonstrate periportal fibrosis with minimal inflammation, indicating a chronic foreign body reaction rather than active immune-mediated hepatitis. Factors such as type of silicone, degree and duration of exposure, time elapsed since rupture, and the individual's immune response may contribute to this variation [8-10,17]. 

Management

All these reported cases of hepatic granuloma are found to have ruptured breast implants. These implants usually need removal to relieve breast pain and rectify asymmetry. Patients are asymptomatic with hepatic granuloma; therefore, no specific treatment is required apart from occasional analgesia, and no routine follow-up is usually required [8-10]. However, these findings are limited by short-term follow-up. Further research is required to understand the prognosis and management of hepatic granuloma as well as the safety profile of silicone breast implants. 

## Conclusions

This case demonstrates a rare systemic complication of silicone breast implants. Although most ruptures are asymptomatic, silicone may spread through blood, lymph, or gravity, resulting in granulomas in distant organs. Liver involvement is uncommon but can resemble malignancy and often needs a biopsy for diagnosis. 

Clinicians should maintain a high index of suspicion for hepatic siliconoma in patients who present with unexplained hepatic lesions or systemic symptoms following breast implant insertion. Imaging modalities such as MRI are critical in assessing both implant integrity and migration patterns, but histopathology remains the gold standard investigation. Increased awareness of this phenomenon can aid timely diagnosis, prevent unnecessary interventions, and guide appropriate patient counseling regarding implant related risks. 
